# Whole Genome Sequencing and Pan-Genomic Analysis of Multidrug-Resistant *Vibrio cholerae* VC01 Isolated from a Clinical Sample

**DOI:** 10.3390/microorganisms11082030

**Published:** 2023-08-07

**Authors:** Vishal Mevada, Rajesh Patel, Pravin Dudhagara, Rajesh Chaudhari, Mustafa Vohra, Vikram Khan, Douglas J. H. Shyu, Yih-Yuan Chen, Dolatsinh Zala

**Affiliations:** 1 DNA Division, Directorate of Forensic Science, Gandhinagar 382007, India; vmevada102@gmail.com; 2 Department of Biosciences, Veer Narmad South Gujarat University, Surat 395007, India; dudhagarapr@gmail.com; 3 School of Applied Sciences and Technology, Gujarat Technological University, Ahmedabad 382424, India; ap_rajeshkumar@gtu.edu.in; 4 Directorate of Medical & Health Services, UT of Dadra & Nagar Haveli and Daman & Diu, Silvassa 396230, India; mustafa_vohra1@yahoo.co.in (M.V.); khandst@rediffmail.com (V.K.); 5 Department of Biological Science and Technology, National Pingtung University of Science and Technology, Neipu, Pingtung 912, Taiwan; dshyu@mail.npust.edu.tw; 6 Department of Biochemical Science and Technology, National Chiayi University, Chiayi City 600, Taiwan; yychen@mail.ncyu.edu.tw

**Keywords:** whole genome sequencing, pan-genomics, AMR, *Vibrio cholerae*

## Abstract

Cholera, a disease caused by the *Vibrio cholerae* bacteria, threatens public health worldwide. The organism mentioned above has a significant historical record of being identified as a prominent aquatic environmental pollutant capable of adapting its phenotypic and genotypic traits to react to host patients effectively. This study aims to elucidate the heterogeneity of the sporadic clinical strain of *V. cholerae* VC01 among patients residing in Silvasa. The study involved conducting whole-genome sequencing of the isolate obtained from patients exhibiting symptoms, including those not commonly observed in clinical practice. The strain was initially identified through a combination of biochemical analysis, microscopy, and 16s rRNA-based identification, followed by type strain-based identification. The investigation demonstrated the existence of various genetic alterations and resistance profiles against multiple drugs, particularly chloramphenicol (*catB9*), florfenicol (*floR*), oxytetracycline (*tet(34)*), sulfonamide (*sul2*), and Trimethoprim (*dfrA1*). The pan-genomic analysis indicated that 1099 distinct clusters were detected within the genome sequences of recent isolates worldwide. The present study helps to establish a correlation between the mutation and the coexistence of antimicrobial resistance toward current treatment.

## 1. Introduction

The pathogen *Vibrio cholerae* is widespread and has long coevolved with humans. It is the agent that causes cholera, a severe intestinal illness that can result in up to 140,000 fatalities and affects between 1 and 5 million people annually [1]. *V. cholerae* carries the cholera toxin and has resulted in several pandemics throughout human history. The seventh *V. cholerae* pandemic continues to this day, and it is even more concerning that it has evolved to present new challenges for treating cholera disease outbreaks [2]. Cholera and similar diarrheal outbreaks can be attributed to both toxigenic and non-toxigenic strains of *V. cholerae*. Over 200 distinct serogroups of *V. cholerae* can be classified based on the antigenic properties of its “O” antigenic lipopolysaccharide [3]. In recent cholera outbreaks, the causative agents have been identified as serogroups O1 and O139, which are known to produce cholera toxins [4]. Insufficient availability of current genome sequences and inadequate monitoring of mutations can lead to the increased probability of bacterial genome diversity and difficulties in identifying diseases and treatment.

The investigation of pathogenicity in strains of *V. cholerae* and their potential for pandemic evolution holds tremendous significance in discerning molecular differentiations in their pathogenicity. *V. cholerae* exhibits various stages of mechanisms in its repertoire for both colonization and infection. The development of resistance in *V. cholerae* species is mainly attributed to genetic recombination, genomic rearrangements, and horizontal gene transfer (HGT), which enable modifications to their genomes [5]. In addition, cholera was thought to be caused only by biotype O1; however, in 1992, a new serogroup of *V. cholerae* called O139 was discovered to be the root of the cholera outbreak in India and Bangladesh [6]. According to the WHO, 94 nations, including India, reported high cholera incidences [7].

The filamentous CTX bacteriophage (CTX), which encodes the cholera toxin, the TCP pathogenicity island that encodes the TCP pili, a colonization factor and receptor for CTX, and toxR, an essential virulence regulatory gene, have all been emphasized in numerous studies as being necessary for pathogenic *V. cholerae* strains [8]. CTX genes are responsible for controlling the primary line, and in the absence of these genes, the infection in the small intestine can be regulated by additional accessory toxic genes such as hemagglutinin protease (HAP), *V. cholerae* protease (PrtV), and a serine protease [9,10]. Concerning the previous incidences, one similarity between the sixth and seventh pandemics has been the CTX virus, which may have given *V. cholerae* access to the CTX toxin [11].

Azithromycin, fluoroquinolones, and tetracycline are just a few antimicrobials successfully used to treat cholera victims over time [12]. However, treatment failures have increased significantly in recent years due to the rapid expansion of the antimicrobial-resistant *V. cholerae*. The present investigation pertains to multidrug-resistant (MDR) *V. cholerae*. Pan-genomic analysis facilitates comprehension of the molecular assessment of particular genes within the genome [13,14]. The extensive investigation has examined the mechanisms underlying *V. cholerae* pathogenicity and its pandemic potential.

## 2. Materials and Methods

### 2.1. Isolation and Antibiotics Susceptibility

A female patient, aged 26, was admitted to Silvassa’s tertiary hospital due to severe symptoms of vomiting and diarrhea. A rectal swab was obtained and enriched with alkaline peptone water under the medical officer’s observation. The selective medium, thiosulfate-citrate-bile-sucrose (TCBS) agar, was used to grow *V. cholerae* from previously collected rectal swabs, followed by biochemical and serological typing. The disc diffusion method was used to evaluate susceptibility to antibiotics like ampicillin, doxycycline, erythromycin, nalidixic acid, chloramphenicol, ciprofloxacin, sulfamethoxazole-trimethoprim, and tetracycline based on the method suggested by Kirby-Bauer [15].

### 2.2. Extraction of Genomic DNA and Whole Genome Sequencing

High molecular weight genomic DNA was extracted from the isolate using a QIAamp DNA Mini Kit (QIAGEN, Hilden, Germany). The genomic DNA was quantified using a Qubit fluorometer (ThermoFisher Scientific, Waltham, MA, USA). The quality of the genomic DNA was assessed using 2% agarose gel [16]. The MinION sequencing library was prepared from 1.5 μg of input DNA as per the manufacturer’s instructions (Oxford Nanopore, Oxford, UK) and multiplexed using 1D Native barcoding kits (EXP-NBD196) followed by a ligation and sequencing kit (SQK-LSK109). The library was loaded on a SpotON flowcell R9.4.1 FLO-MIN106 and sequenced for 72 h on the MinION Mk1C device. The fast5 data from MinKNOW were converted to fastq format using the Guppy base caller in fast mode on a MinIT device (Oxford Nanopore, Oxford, UK). The fastq reads were demultiplexed using qcat v1.0.1.3 [17]. All short-read sequences were first considered to detect the contamination of the other organisms. The Kraken 2.0 tool was used to detect the source of sequences based on k-mer to detect the contamination at the stage of isolation as discussed by Luc Cornet and Denis Baurain [18].

### 2.3. Type Strain-Based Identification, MLST, and Whole Genome Annotation

The genome assembly was constructed using the Canu assembler on the Galaxy Australia webserver (https://usegalaxy.org.au/, accessed on 30 June 2022). The TrueBac ID system and Type (Strain) Genome Server (TYGS) were used to carry out a whole genome-based taxonomic analysis [19,20]. Based on selected closely related type strains were further compared with the MASH algorithm [21]. All pairwise comparisons among the collection of genomes for the phylogenomic inference were carried out using GBDP, and precise intergenomic distances were inferred using the algorithm “trimming” and the distance formula followed by phylogenetic inference using FASTME 2.1.6.1 after post-processing [22,23]. MLST v2.0 (https://cge.food.dtu.dk/services/MLST, accessed on 30 June 2022) was utilized to validate further species identification based on type strain-based identification [24]. Whole genome annotation of the entire genome was accomplished with the help of Prokka 1.11 [25]. The output was used for the Roary-based pan-core genome analysis in the later analysis stage. The RAST and PATRIC server was used for Gene-finding, annotation, and functional categorization [26,27]. Using the RAST annotation server, 16S rRNA genes of high quality were retrieved from the investigated genomes, and the sequences were aligned using MUSCLE. Finally, a UPGMA tree was created with MEGA11 with a bootstrap value of 500 [28].

### 2.4. Detection of Mobile Genetic Elements

*V. cholerae* has been observed to exhibit a significant proportion of mobile genetic elements (MGEs), including prophage sequences, insertion sequences, CRISPR-Cas systems, and plasmids [29]. The ICEberg v.2.0 tool was used with default parameters to detect the presence of MGEs in the isolated strain [30]. The online tools PHASTER and ISfinder were utilized to detect the prophage sequences and insertion sequences, respectively, in the genome of *V. cholerae* VC01 [31,32], while the identification of plasmids in the genome of isolated strains was compared and analyzed using PLSDB and NCBI-Plasmidfinder [33].

### 2.5. Identification of the Antimicrobial Resistance (AMR) Gene

Four approaches determined the presence of acquired resistance genes: Abricate (https://github.com/tseemann/abricate, accessed on 30 June 2022), One-Codex (https://www.onecodex.com, accessed on 30 June 2022), Blastn against Virulence factor database(VFDB) [34], and Resistance Gene Identifier (RGI) [34]. All methods utilized the default parameters with lengths ≥ 90% and identities of ≥60 except Abricate. In Abricate, the genome sequences were screened against all databases to get a detailed comparative analysis of resistance.

### 2.6. Pan-Genome Analysis

Using OrthoFinder 1.1.8, ortho-groups were compiled, and trees based on the presence–absence of ortho-group members were created to understand the highly diverse type of Genomes [35]. The Roary tool was used to perform a pan-genome analysis of 20 genome sequences reported from nations like Bangladesh, Haiti, and India [36]. Phandango [37] and recommended R scripts were used to view the resulting output graphs using RAxML [38].

## 3. Results

### 3.1. Isolation and Antimicrobial Susceptibility

A rectal swab sample was collected from patients with acute diarrhoeal disease in a tertiary hospital at Silvassa (India) showing sporadic cholera cases from hospitalized patients. The antimicrobial drug susceptibility exhibits a strain showing agglutination with polyvalent O1 antisera. The preliminary test results based on agglutination show similarity to the *V. cholerae* O1 biotype. Irrespective of their biotype, the isolated *V. cholerae* VC01 strain was resistant to amikacin, gentamicin, co-trimoxazole, and nalidixic acid, while retaining susceptibility towards tetracycline, chloramphenicol, and trimethoprim as observed in the organism. Only some genome sequencing studies of V. cholerae are available from the western region of India. To understand the changes in the genome sequence of the present isolate, we carried out whole genome sequencing of one isolated organism to find differences in the genomic level to compare genomes during pre- and post-COVID-19 situations.

### 3.2. Genome Assembly and Identification

**Figure 1:**
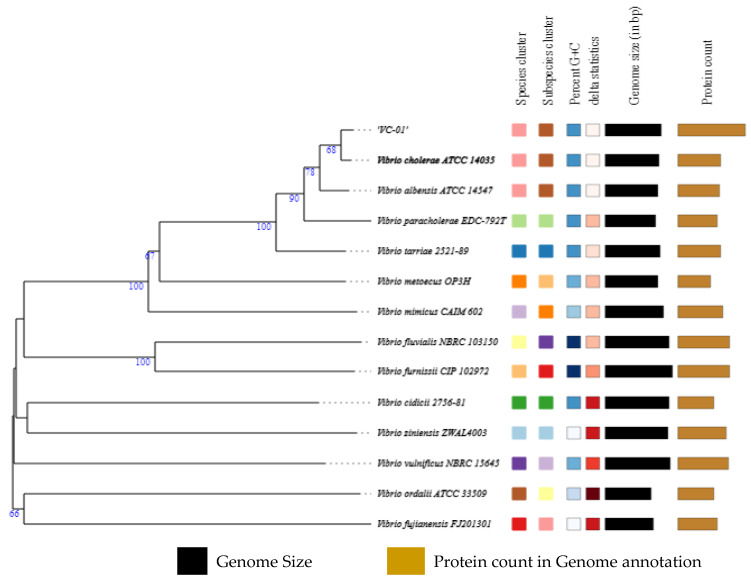
Type Strain-based maximum likelihood organisms reported compared to isolated *Vibrio cholerae* VC-01 Strain.

The MiniONT sequencer originally produced the raw sequences in fastq format. The results indicated no presence of contaminating sequences from other organisms. The Flye Assembler assembled two contigs with a total genome size of 4,101,607 base pairs having a 47.47% GC ratio observed. The results of QUAST indicated the 10× Genome Coverage for both assembled chromosomes. The BUSCO tool showed 82.43% genome sequence completeness based on similarity. Based on Type (Strain) Genome Server (TYGS) results, 14 strains were found with a closely related genome sequence for the isolated organism. The nearest match observed was to the *V. cholerae* ATCC 14035 strain, followed by *Vibrio albensis* ATCC 14547, *Vibrio paracholerae* EDC-792T, and *Vibrio tarriae* 2521-89 (Figure 1).

According to the findings, the isolated strain had a significant protein sequence annotation and was closely related to the *V. cholerae* ATCC 14035 strain. These results also show consecutive confirmation using biochemical analysis performed for the isolated strain. Based on the results, the isolate underwent MLST profiling which confirmed its close clustering with *V. cholerae* (Table 1).

### 3.3. Genome Annotation

**Figure 2:**
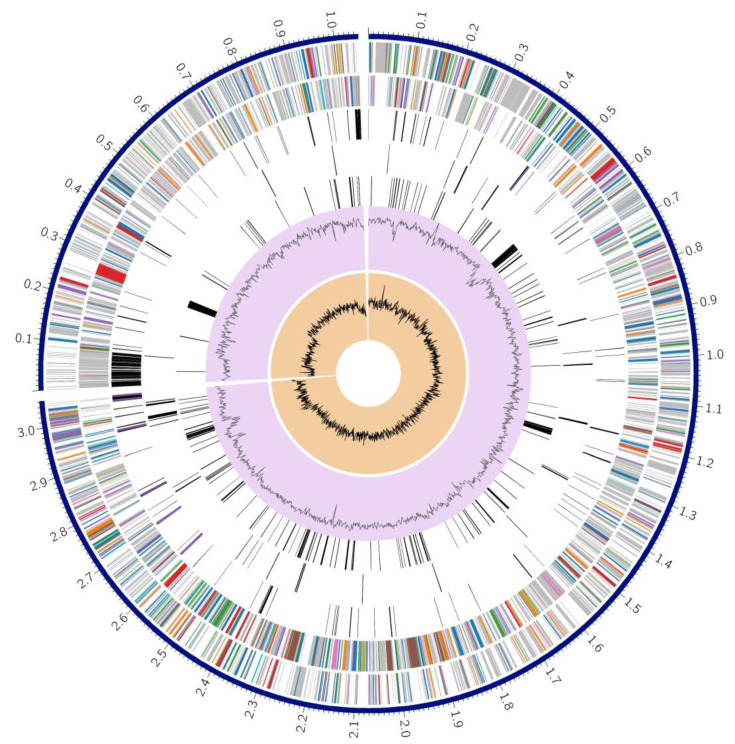
Complete genome annotation of *Vibrio cholerae* VC01. (The components mentioned in Figure 2, arranged in a hierarchical order from the outermost to the innermost rings, comprise the contigs, CDS sequences located on the forward strand, CDS on the reverse strand, RNA genes, CDS with homology to known antimicrobial resistance genes, CDS with homology to known virulence factors, as well as the GC content and GC skew).

Two distinct approaches, Prokka and RAST, were utilized for the annotation of the genome. Each of these methods possesses unique advantages. Prokka-based annotation was used to analyze pan-genomes, whereas RAST-based study provided annotation based on sub-systems. A genome analysis using Prokka produced the following findings: 5310 CDS, 5520 Gene, 96 tRNA molecules, seven rRNA, 740 pseudogenes, and 914 hypothetical genes. The hypothetical proteins were mapped to their functional assignment using the PATRIC server [27]. The proteins with functional assignments included 1614 proteins with Enzyme Commission (EC) numbers [39], 1324 proteins with Gene Ontology (GO) assignments [40], and 1136 proteins that were mapped to KEGG pathways [41]. PATRIC annotation includes two types of protein families, and this genome has 5486 proteins that belong to the genus-specific protein families (PLFams) and 5579 proteins that belong to the cross-genus protein families (PGFams) (Table 2). A complete circular graphical display of the distribution of the genome annotations is provided in Figure 2.

**Figure 3:**
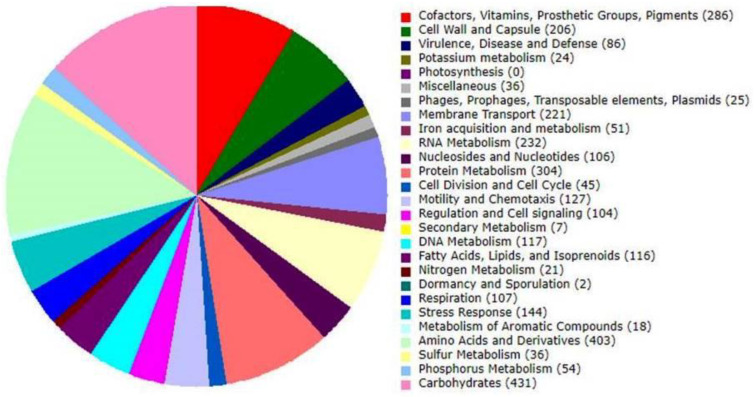
Sub-system-based annotation.

**Figure 4:**
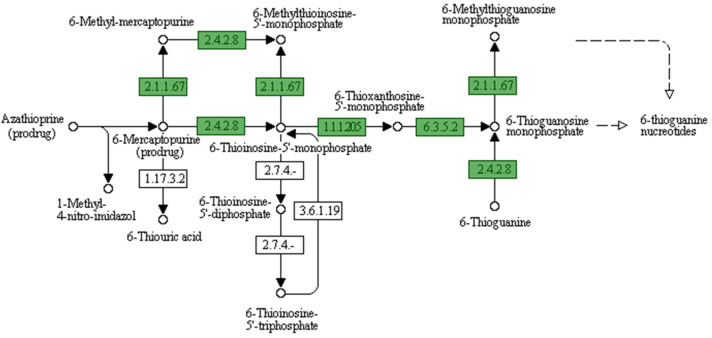
Azathioprine and 6-Mercaptopurine metabolism pathway.

**Figure 5:**
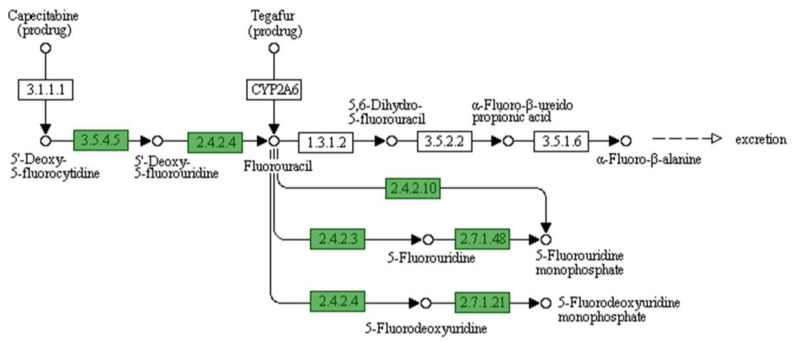
Fluorouracil metabolism pathway.

In total, 25 features were observed for phages, prophages, transposable elements, and plasmids related to sub-systems-based annotation (Figure 3). KEGG and GO ontology-based analysis also show several precursor molecules for drug metabolism, eventually resulting in antimicrobial resistance in *V. cholerae*. From the current annotation, we identified two metabolic pathways that have functional importance to drug metabolism. One pathway is Azathioprine and 6-Mercaptopurine metabolism (Figure 4), and the second is the Fluorouracil metabolism pathway (Figure 5). However, the substrate of the pathway was not directly used for treating cholera; their importance was observed for cholera disease progression. A drug called azathioprine (AZA) is used to manage and treat active rheumatoid arthritis (RA), as well as to avoid kidney transplant rejection [42], Mercaptopurine and 5-Fluorourasil are extensively used for the chemotherapy treatment of cancer patients [43]. The metabolic pathway to degrade 5-Fluorourasil also damages intra-genus and inter-species quorum-sensing autoinducers, allowing the growth of *V. cholerae* and dispersal into the patient’s body.

### 3.4. Identification of Antibiotic Resistance Genes

**Figure 6:**
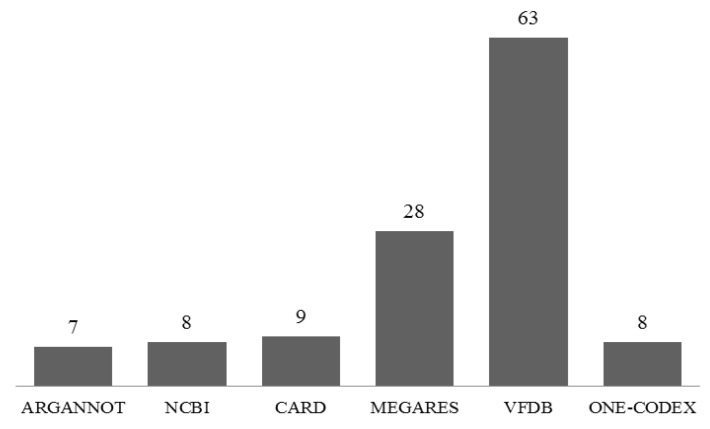
Antibiotic resistance/virulence factors evaluated against various databases.

Antibiotic resistance is a severe problem with *V. cholerae*. Using the Virulent Factor Database (VFDB) as a reference database, Abricate’s findings revealed 63 antibiotic resistance genes, followed by 28 from the Megares database using 75% identity/coverage. The analysis was also carried out for CARD, the NCBI antimicrobial resistance database, and the Argannot database and showed the presence of 9, 8, and 7 genes, respectively (Figure 6). The One-codex analysis revealed the presence of *strB* and *cat9B* genes associated with resistance to aminoglycosides and Chloramphenicol. At the same time, six genes (*emrD, vcaM, vceA, vceB, vcmA*, and *vcrM*) were identified as conferring resistance to the multidrug efflux drug class. Aminoglycoside, diaminopyrimidine, carbapenem, macrolide, fluoroquinolone, penam, phenicol, elfamycin, and multidrug efflux-based resistance are the main categories of antibiotic resistance that have been reported in the genome sequence of an isolated organism (Table 3).

A resistance gene was detected that belongs to *Escherichia coli* EF-Tu mutants conferring resistance to pulvomycin. This resistance gene could be horizontally transferred to *V. cholerae*. An R234F SNP was also detected in this resistance gene family; hence, the elfamycin resistance can be seen in the VC-01 strain. In addition, the homologous sequences for several organisms, including *V. cholerae* O1 biovar El Tor str. N16961, *Yersinia enterocolitica* subsp. enterocolitica 8081, *Legionella pneumophila* subsp. pneumophila str. Philadelphia 1, *Haemophilus influenzae* Rd KW20, and *Neisseria meningitidis* MC58 were reported upon comparison with the Virulence Factor Database (VFDB).

### 3.5. Identification of Integrative Conjugative Element (ICE)

**Figure 7:**
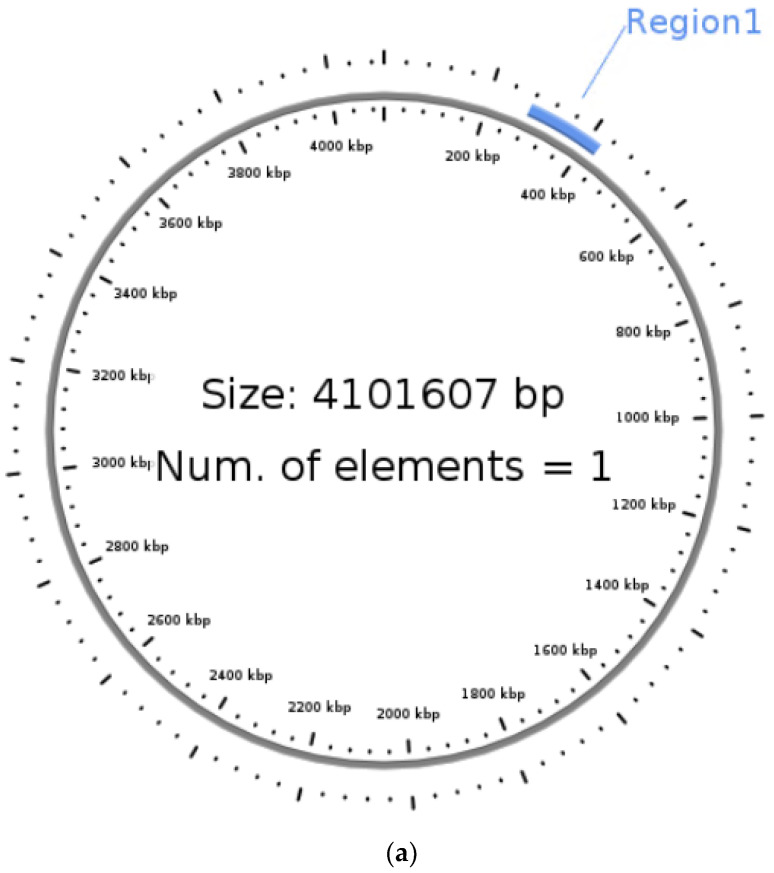

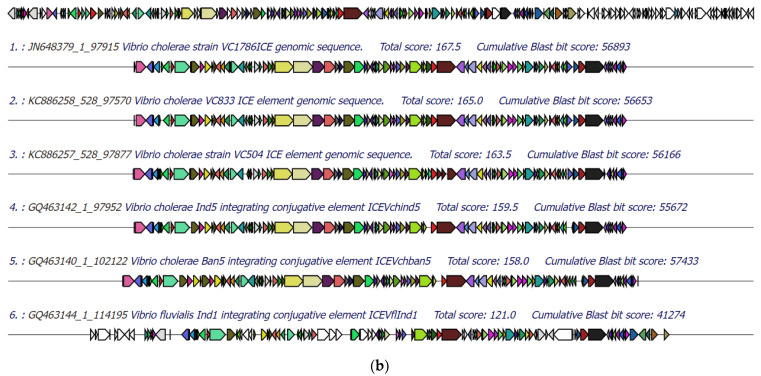
Integrative Conjugative Element (ICE) in the genome sequence of *Vibrio cholerae* VC01 (Region 1 specifies the location of ICE identified in the circular genome). (**a**) Circular view of the genome representing ICE region. (**b**) Alignment view of whole genome sequence using ICEberg database.

ICEfinder predicted an ICE region on the analyzed genome sequence at 278,238–424,664 nucleotides covering 146,427 base pairs (Figure 7) as operons encoding a type IV secretion system (T4SS). Among the 198 ORF sequences, 28 were found to be similar to related species from the ICEberg database. The best match was observed with *V. cholerae* strain VC1786ICE (NCBI Accno: CP028827.1) with a maximum score of 167.5, followed by 165 for *V. cholerae* VC833 ICE element genomic sequence (Genbank Accno: KC886258.1) and 163.5 for *V. cholerae* strain VC504 ICE element genomic sequence (Genebank Accno: KC886257.1).

### 3.6. Identification and Comparison of Prophage Sequences

The current study aims to identify the presence of phage sequences within the genetic material of an isolated strain by utilizing the PHAge Search Tool Enhanced Release (PHASTER) [31]. The PHASTER analysis identified four prophage regions: one intact region and three incomplete regions (Table 4). All four regions were found to be distributed throughout the genome sequence. This may indicate variations of gene transfer recorded at different intervals. The prophage region predicted to be intact exhibits a significant similarity with the prophage sequence derived from *Vibrio phage* CTXphi (NCBI Acc No: NC_015209.1). However, three incomplete prophage regions belong to *E. coli* and *P. aeruginosa*. The co-morbid presence of *V. cholerae* with other filamentous organisms from clinical samples also reported such transfer [44]. Despite being a relatively underexplored phenomenon, this occurrence may offer valuable insights into the evolution and the emergence of multidrug resistance in *V. cholerae*-like organisms.

### 3.7. Pan-Core Genome Analysis

**Figure 8:**
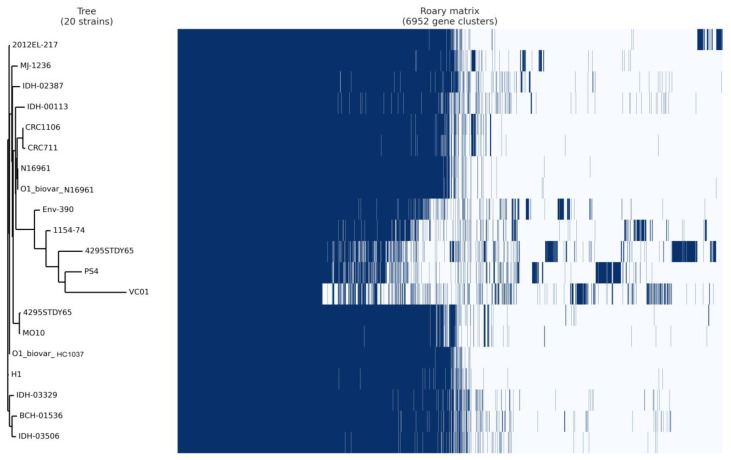
Pan-core genome analysis of selected genomes.

A total of 20 complete genomes were selected based on the similarity, occurrence, and literature survey for epidemiological importance (Table S1). These genomes were annotated using Prokka and analyzed using the Roary tool for a comprehensive analysis of pan-core genomics for pre- and post-COVID-19 implications. The Roary-based analysis findings showed 6952 clusters, including 1882 core clusters, 805 soft-core gene clusters, 3002 cloud clusters, and 1297 shell clusters of genes. Hence, the core-to-pan ratio was 54%, indicating significant gene conservation across the entire genome sequence used in the current study. The isolated *V. cholerae* VC01 strain is related to the *V. cholerae* PS4 strain as a distinct group, according to phylogenetics based on the pan-genomic study (Figure 8). Previous research indicated that the V. cholerae PS04 strain was isolated from puffer fish as a non-sucrose fermenting organism [45]. However, the strain harbored the T3SS-1 system, conferring cytotoxicity on the patient’s body.

## 4. Discussion

In recent years, the incidence of *V. cholerae* has gradually increased after the COVID-19 situation. This is attributed to the growth of global tourism, which will increase the likelihood of individuals being exposed to *V. cholerae* outside their home countries, leading to the development of carriers or new hosts. Despite the occurrence of seven pandemics and numerous epidemics worldwide, efforts to combat cholera remain ongoing. The disease is characterized by profuse watery diarrhea that can lead to dehydration and hypovolemic shock. The primary mode of transmission through the fecal-oral route also is a source of infection [46]. During phase 1 of COVID-19, people were more conscious regarding hygienic conditions. However, as things returned to normal and limitations eased, mass gatherings and travelling once again became common, leading to factors in the spread of diseases like cholera in subsequent stages. The emergence of antibiotic-resistant strains of *V. cholerae* has posed a significant challenge in the future.

This present study’s whole genome sequencing and comparative genomics methodology provide insight into the diversity of the V. cholerae strain VC01 isolated from clinical samples. A similar study was conducted to characterize the genome for pathogenic and non-pathogenic *V. cholerae* isolated from migratory birds at Dali Nouer Lake in China [4]. Our findings enhance and expand our understanding of the variations in the evolution of virulence determinants, antibiotic-resistant determinants, the existence of integrative and conjugative elements (ICE), mobile genetic elements (MGE), and pan-genome analysis in clinical *V. cholera* isolates. The integrative and conjugative element (ICE) is a critical member of the bacterial mobile genetic elements, which is integrative to the bacterial chromosome, encodes fully functioning conjugation machinery, and is thus self-transmissible between bacterial cells. The genes present in ICEs, which encode for both antibiotic resistance determinants and virulence factors, can provide the host with selective advantages. ICEfinder offers an efficient way to detect the presence of ICE sequences by comparison with its database [30]. Earlier research has documented the identification of T4SS-like elements through in silico analysis in both environmental and clinical isolates of *V. cholerae* O1 and non-O1/non-O139 from the Indian subcontinent and South America, as well as in Shewanella sp. from North America [47]. However, these elements appear to lack antibiotic-resistance genes. Later on, with the emergence of IDH-03944, a strain of *V. cholerae* O44 that exhibits resistance to clotrimoxazole has been linked to MGIVchHai6 T4SS. The comparative study regarding the ICE and its importance previously revealed that the genome of *V. cholerae* O1 isolates responsible for the cholera outbreak in Haiti in 2010 contained ICEVchHai1, which carried auxiliary support conferring resistance to trimethoprim (*dfrA1*), sulfamethoxazole (*sul2*), streptomycin (*strAB*), and chloramphenicol (*floR*) [48]. Similar sets of antimicrobial resistance genes have been documented in ICEs in India (1994–2005), Bangladesh (1998), Nepal (1994), and Nigeria (2010) [49].

The detected prophage sequence from an isolated organism was found in a similar sequence of Phage CTX observed in *V. cholerae* O1 [50]. This integration of Phage CTX into the genome might be due to gene transfer between phage CTX and the RS1 region of genome. The point mutation in the cholera toxin gene ctxB also promotes new CTX prophages into the genome sequence. Although exploring prophage sequences in various organisms has been limited, the prophage sequences in *V. cholerae* genomes were thoroughly investigated. The *V. cholerae* O1 serogroup classical biotype strains possess the cholera-toxin encoding phage CTX(cla) specific to their biotype, while the El Tor biotype strains carry CTX-1 [51]. However, the CTXΦ phage in the classical biotype is usually integrated solitarily or with a truncated copy.

Generally, Norfloxacin, trimethoprim-sulfamethoxazole (TMP-SMX), ciprofloxacin, and azithromycin have been used to treat cholera. The World Health Organization (WHO) recommends the management of cholera with oral rehydration salts in combination with antibiotics such as streptomycin, aminoglycosides, trimethoprim, fosfomycin, fluoroquinolones, sulphonamides, chloramphenicol/florfenicol, and tetracyclines [52]. Usually, bacterial species including *V. cholerae* can acquire resistance to antimicrobial compounds, antibiotic target replacement, and antibiotic target protection, and prompt inactivation of the antibiotic by hydrolysis, preventing access to the target site by changing membrane permeability and actively exporting antibiotics from bacterial cells [48]. In the present study, the isolate was resistant to several antibiotics, including aminoglycosides, carbapenems, and macrolides, leading to the emergence of the multidrug-resistant strain of *V. cholerae*. Additionally, the isolate was resistant to pulvomycin, which was horizontally transmitted to *V. cholerae*. A similar antibiotic resistance mechanism was previously reported in the Uganda study during 2014–2016 [53].

Although it is presently difficult to predict, the effect of treatment for infectious disease will affect antimicrobial resistance. However, most studies show extensive empirical use in contrast to comparatively infrequent bacterial co-infection and secondary infections [54]. Exposure to antimicrobial agents contributes to the escalation of microbial resistance [55]. Furthermore, prolonged hospitalization in the intensive care unit intensifies the susceptibility to infections caused by multidrug-resistant organisms [56]. The use of antibiotics may also affect the microbial flora in the digestive tract, promoting the development of resistant strains of several Infectious agents [57]. However, such a phenomenon was also reported earlier, such as gene swapping [58], quorum-sensing-based horizontal gene transfer [59], and SOS response [60], promoting multidrug resistance in *V. cholerae*. Antimicrobial resistance in *V. cholerae* is also known to be due to the acquisition of resistance genes from closely or distantly related microbial species through horizontal gene transfer [61]. Inadequate sanitation and water contamination were also discovered to have a rapid horizontal gene transfer in *V. cholerae* [62,63].

Based on pan-genomic analysis, out of 1099 unique clusters, 22 were identified as having a role in CRISPR (04), drug resistance (07), phage sequences (02), and toxin-related functions (09). The unique clusters (Table 5) were reported in the genome sequence of the isolated V. cholerae VC01 in the present study. The gene/clusters reported as unique were previously reported from organisms like *Actinobacillus pleuropneumoniae*, *Aggregatibacter actinomycetemcomitans*, *Aliivibrio wodanis*, *Bacillus subtilis*, *Escherichia coli*, *Kingella kingae*, *Pseudoalteromonas* sp., *Salmonella enterica*, *Staphylococcus aureus*, *Vibrio* spp. Similarly, Verma et al. (2019) also reported the cross-species transfer of resistance genes to *V. cholerae* genomes from 2008 to 2015 [61]. Pan-genome analysis showing a slight variation in the number of accessory and unique genes in the isolated strain revealed alteration compared to selected genomes used in present study. Additionally, the size of the pan-genome is consistently increasing in the isolated collection. This variation may be attributed to the development of mechanisms for acquired antimicrobial resistance in strains from the medical environment or the proliferation of new genes.

Over time, *V. cholerae* resistance to major classes of antibiotics is concerning. An example is the incidence of quinolone resistance genes in *V. cholerae* O139 strains from China that were conjugative and contributed due to a CIP-resistant plasmid similar to that observed in *V. vulnificus* [84]. Additionally, mobile genetic elements, particularly plasmids, have significantly contributed to the spread of antimicrobial resistance. Some studies also found plasmid from other strains of *V. cholerae* [85]. All these incidences were already reported in the past, supporting our finding regarding the antimicrobial resistance from the isolated strain. In contrast to these, researchers from Kolkata, India, also reported a Non-O1/non-O139 non-toxigenic *V. cholerae* strain having resistance towards more than three groups of antibiotics using genome analysis [86]. Such types of strategic analysis can simply be made more efficient through the use of complete genome sequencing and molecular characterization to track successive linked outbreaks.

In August 2020, the first round of the OCV campaign was done in the most affected districts of the Littoral, South, and Southwest regions [87]. The number of cholera cases was considerably reduced after this first round. It is unknown whether the *V. cholerae* vaccine can show the same scenario in the future. However, the antibiotic susceptibility data of publicly available genomes were not included in the analysis as the data were not available. Hence, there is a need for genome-based surveillance from all other currently isolated strains that might provide more insight into such antibiotic resistance. The present study provides insight into whole genome sequencing of such isolates from the patient and also can be replicated for future isolates for tracking cholera like infectious diseases.

## 5. Conclusions

Recently, due to COVID-19 implications, the emphasis on infectious diseases such as cholera shifted, resulting in high fatality rates worldwide due to co-morbid infection with *V. cholerae.* Pan-genomic profiling offers valuable insights into the presence of additional sequences in *V. cholerae* through gene transfer, which can confer resistance to multiple antibiotics. The findings demonstrated the need to explore alternative therapeutic approaches for managing multidrug-resistant cholera. In addition, it is beneficial to perform re-efficacy testing on presently utilized vaccines against *V. cholerae.* This may offer explicit knowledge of the possible existence of epitopes derived from other organisms as a potential site for the development of vaccines. The presence of antibiotic resistance genes in the whole genome and in vitro study of susceptibility towards similar antibiotics shows a correlation of gene expression in the isolated strain. This research has generated interest in further genomic profiling and understanding the dissemination and multidrug resistance using whole genome sequencing of *V. cholerae*. India, like other Asian countries, carries more asymptomatic V. cholerae incidences every year, leading to increased mutation of multidrug resistance. The concurrent whole genome sequencing provides a detailed understanding of the dissemination and multidrug resistance in such infectious agents.

## Figures and Tables

**Table 1:** Multilocus sequence typing (MLST) profile of *Vibrio cholerae* VC01.

Locus	Identity	Coverage	Alignment Length	Allele Length	Gaps	Organism
*adk*	100	100	416	416	0	*V. cholerae*
*gyrB*	99.768	99.768	431	431	1	
*mdh*	99.0499	99.0499	421	421	4	
*metE*	100	100	591	591	0	
*pntA*	100	100	431	431	0	
*purM*	99.5798	99.5798	476	476	2	
*pyrC*	99.5546	100	449	449	0	

**Table 2:** Protein feature analysis of *Vibrio cholerae* VC01 using PATRIC tool.

No	Protein Feature	Count
1	Hypothetical proteins	812
2	Proteins with functional assignments	4870
3	Proteins with EC number assignments	1614
4	Proteins with GO assignments	1324
5	Proteins with Pathway assignments	1136
6	Proteins with PATRIC genus-specific family (PLfam) assignments	5486
7	Proteins with PATRIC cross-genus family (PGfam) assignments	5579

**Table 3:** Resistance against different drug classes, AMR gene families, and resistance mechanisms.

Resistance Mechanism	Gene	AMR Gene Family	Drug Class	Identity (%)	Coverage (%)
Antibiotic efflux	*CRP*	resistance-nodulation-cell division (RND) antibiotic efflux pump	macrolide antibiotic, fluoroquinolone antibiotic, penam	95.24	100
Antibiotic inactivation	*APH (3″)-Ib*	APH (3″)	aminoglycoside	98.81	94.38
	*catB9*	chloramphenicol acetyltransferase (CAT)	phenicol antibiotic	99.52	99.52
	*Vibrio cholerae varG*	Subclass B1 *Vibrio cholerae* varG beta-lactamase	carbapenem	99.7	84.87
Antibiotic target alteration	*E. coli EF-Tu*	elfamycin resistant EF-Tu	elfamycin antibiotic	87.28	96.33
	*dfrA1*	trimethoprim-resistant dihydrofolate reductase dfr	diaminopyrimidine	99.36	100
Antibiotics resistance	*SUL2*, *DFRA1*	dihydropteroate synthase (DHPS)	Sulfonamide and trimethoprim-resistant dihydropteroate synthase Sul2	99.14	98.9
Efflux Regulator	*EMRD*	Drug and biocide resistance	Multidrug efflux	100	100
	*VCEA*	Drug and biocide resistance	Multidrug efflux	98.24	99.75
	*VCEB*	Drug and biocide resistance	Multidrug efflux	98.31	99.93
	*VCER*	Drug and biocide resistance	Multidrug efflux	99.83	99.83
	*VCMA*	Drug and biocide resistance	Multidrug efflux	99.71	99.86

**Table 4:** Prophage regions in *Vibrio cholerae* VC01.

Region	Length	Completeness	Score	Total Proteins	Region Position	Most Common Phage	GC (%)
1	10.7 Kb	incomplete	60	14	315,989–326,748	PHAGE_Escher_500465_1_ NC_049342(7)	47.13
2	12.1 Kb	intact	150	30	966,779–978,912	PHAGE_Vibrio_CTX_ NC_015209(13)	45.67
3	12.7 Kb	incomplete	50	26	1,339,522–1,352,283	PHAGE_Pseudo_JBD18_ NC_027986(3)	42.96
4	20.6 Kb	incomplete	60	13	3,749,246–3,769,881	PHAGE_Escher_RCS47_ NC_042128(2)	45.76

**Table 5:** Unique clusters identified from *Vibrio cholerae* VC01.

No	Cluster	Gene	Annotation	Function	Organism	Reference
1	casC	casC	CRISPR system Cascade subunit CasC	CRISPR	*Vibrio* spp.	[29]
2	csy3	csy3	CRISPR-associated protein Csy3	CRISPR	*Aliivibrio wodanis*	[64]
3	group_1026	qorA	Quinone oxidoreductase 1	Resistance	*Staphylococcus aureus*	[65]
4	group_1096	zot	Zona occludens toxin	Toxin	*Vibrio parahaemolyticus*	[66]
5	group_1408	pspA	Phage shock protein A	Phage	*Vibrio cholerae*	[67]
6	group_1432	bcr	Bicyclomycin resistance protein	Resistance	*Escherichia coli K12*	[68]
7	group_146	endo I	Chitodextrinase	Resistance	*Pseudoalteromonas* sp. *P1-9*	[69]
8	group_1966	tcpE	Toxin coregulated pilus biosynthesis protein E	Toxin	*Vibrio cholerae*	[70]
9	group_2021	group_2021	IS3 family transposase ISVpa4	Resistance	*Escherichia coli*	[71]
10	group_307	intA	Prophage integrase IntA	Phage	*Salmonella enterica*	[72]
11	group_3154	ccdB	Toxin CcdB	Toxin	*Vibrio fischeri*	[73]
12	group_33	rtxA	Multifunctional-autoprocessing repeats-in-toxin	Toxin	*Kingella kingae*	[74]
13	group_373	mepA	Multidrug export protein MepA	Resistance	*Staphylococcus aureus*	[75]
14	group_3960	higB-1	Toxin HigB-1	Toxin	*Vibrio cholerae*	[76]
15	group_3962	higA-1	Antitoxin HigA-1	Toxin		[76]
16	group_442	bmr3	Multidrug resistance protein 3	Resistance	*Bacillus subtilis*	[77]
17	group_461	ltxB	Leukotoxin export ATP-binding protein LtxB	Toxin	*Aggregatibacter actinomycetemcomitans*	[78]
18	group_655	fruB	Multiphosphoryl transfer protein	Resistance	*Vibrio cholerae Pst2*	[79]
19	group_76	apxIB	Toxin RTX-I translocation ATP-binding protein	Toxin	*Actinobacillus pleuropneumoniae*	[80]
20	mazF	mazF	Endoribonuclease toxin MazF	Toxin	*Escherichia coli*	[81]
21	ygbF	ygbF	CRISPR-associated endoribonuclease Cas2	CRISPR	*Escherichia coli*	[82]
22	ygbT	ygbT	CRISPR-associated endonuclease Cas1	CRISPR	*Escherichia coli K12*	[83]

## Data Availability

Whole genome sequences have been deposited and are publicly available on NCBI with Bioproject ID: PRJNA853502 (https://www.ncbi.nlm.nih.gov/bioproject/PRJNA853502) (accessed on 30 June 2022). and SRA ID: SAMN29394033 (https://www.ncbi.nlm.nih.gov/sra/SRX15925945) (accessed on 15 July 2022).

## Supplementary Materials

The following supporting information can be downloaded at: https://www.mdpi.com/article/10.3390/microorganisms11082030/s1, Table S1. List of genomes used for pan-genomic analysis.
